# Mediastinal Tuberculous Lymphadenitis Presenting as a Mediastinal Mass with Dysphagia: A Case Report

**Published:** 2011-09-25

**Authors:** F. Sahin, P. Yildiz

**Affiliations:** 1 Pulmonologist, Department of Chest Diseases, Yedikule Chest Diseases and Thoracic Surgery Research and Training Hospital, İstanbul, Turkey; 2 Associate Professor, Department of Chest Diseases, Yedikule Chest Diseases and Thoracic Surgery Research and Training Hospital, İstanbul, Turkey

**Keywords:** Mediastinal Lymph Node, Thoracotomy, Tuberculosis

## Abstract

Mediastinal tuberculous lymphadenitis is a rare disease in adults. Dysphagia as the accompanying symptom is even a rarer manifestation. Cases with esophageal symptoms may present as esophageal ulceration, mucosal or submucosal mass, fistula or sinus formation, extrinsic compression or displacement of the esophagus. Extrinsic compression may radiologically or endoscopically present as a submucosal tumor. Our case is a 30-year-old woman with dysphagia for a month. Extrinsic compression was seen endoscopically on the mid-esophagus. On thoracic CT and MRI images, a multiloculated cystic/necrotic mass 5.5×4.8×3.1 cm in size consisting of multiple septa was located subcarinally in the middle mediastinum. In Wang needle aspiration, a mucopurulaent liquid was aspirated from the subcarinal localization by bronchoscopy. Diagnostic thoracotomy was carried out because histological and bacteriological examinations were not diagnostic. It was reported as tuberculous lymphadenitis pathologically. The control thoracic CT performed after antituberculous treatment showed complete regression of the mass. We herein report a rare form of tuberculous lymphadenitis.

## Introduction

Dysphagia due to mechanical obstruction of the esophagus is often related to a malignant esophageal disease. Benign lesions causing such obstructions are quite rare and they tend to manifest themselves as intramural tumors or extrinsic compressions. Lymphadenitis due to mediastinal tuberculosis is rarely seen in adults; dysphagia caused by this disease is much more rare.[1] Dysphagia due to tuberculosis is encountered in high tuberculosis incidence regions and in patients with supressed immunity.[2] A 30-year-old female patient admitted with dysphagia diagnosed as tuberculous lymphadenitis on diagnostic thoracotomy is presented in this case report.

## Case Presentation

A 30-year-old female patient was admitted to an internal medicine clinic with the complaint of difficulty on swallowing for a month and endoscopy revealed external compression on the middle part of the esophagus. She was referred and admitted since the chest MRI revealed a subcarinal mass in the middle mediastinum. The past medical history revealed a surgery for hydatic liver cyst.

The physical examination revealed a blood pressure of 110/70 mmHg, pulse of 88/ min and a respiratory rate of 18/min. All system findings were normal. Laboratory findings revealed minimal hypochromic microcytic anemia in complete blood count. Biochemical tests were normal. The erythrocyte sedimentation rate was 32/h. Arterial blood analysis revealed slight hypoxemia. Lung function tests revealed medium obstruction and restriction (FEV1: 63%, FVC: 68%, FEV1/FVC: 81%).

**Fig. 1:**
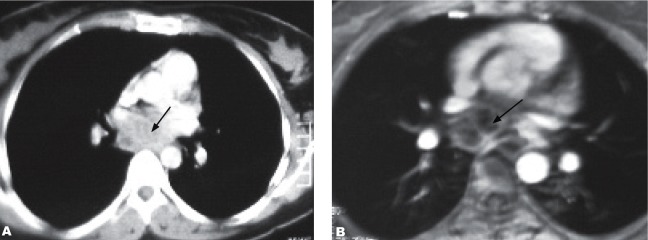
A 30-year-old female patient with mediastinal tuberculous lymphadenitis presenting as a mediastinal mass with dysphagia. A. Thoracic CT shows a 5.5×4.8×3.1 cm mass with subcarinal localization in the middle mediastinum with a multilocular cystic/necrotic appearance and multiple septa. B. MRI shows a subcarinally located 5.5×4,8×3.1 cm mass in the middle mediastinum with a multilocular cystic/necrotic appearance and multiple septa.

**Fig. 2:**
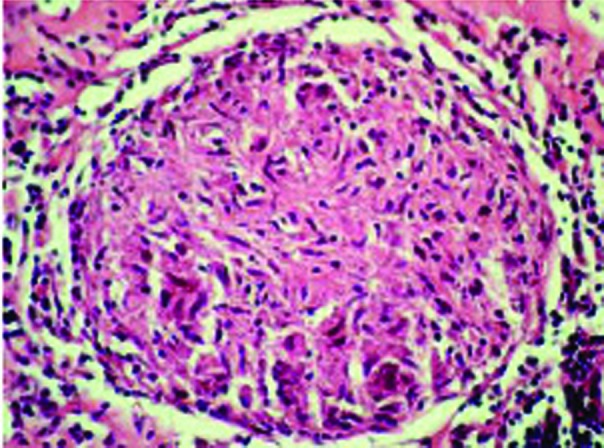
The pathologic examination of the specimen demonstrates granulomatous inflammation (H-E´400).

The chest X-ray was normal. Lung CT and MRI examinations showed a subcarinally located 5.5×4.8×3.1 cm mass in the middle mediastinum, which had a multilocular cystic/necrotic appearance with multiple septa (Fig. 1). No endobronchial lesion was detected on bronchoscopic examination. A mucopurulent fluid was obtained with transbronchial needle aspiration performed with a Wang 122 needle through the subcarinal region. As cytological and bacteriological examination of the fluid did not yield an exact diagnosis, diagnostic thoracotomy was performed. Histopathological examination of the material obtained by thoracotomy revealed tuberculous lymphadenitis (Fig. 2). Lesions improved completely with a full dose combined treatment with four anti-tuberculosis medications.

## Discussion

Mediastinal tuberculous lymphadenitis cases with esophageal symptoms may admit in forms of esophageal ulceration, mucosal or submucosal mass with ulceration, sinus or fistula formation, external compression or dislocated esophagus. In case of extreme external compression, the disease may be presented as submucosal tumor radiologically or endoscopically.[3] Our case was examined for dysphagia in the gastroenterology clinic and the esophagus was narrowed because of external compression. Thoracic MRI revealed that the pathology causing compression was a multiloculated cystic mass in the subcarinal region. Further examinations revealed that the mass was actually a tuberculous lymphadenitis manifesting itself as a mediastinal mass.

Differential diagnosis of formations radiologically presenting as a mediastinal mass is quite important. Generating a differential diagnosis for a mediastinal mass starts with a classification scheme. The system used by Felson divides the mediastinum into anterior, middle and posterior compartments by drawing a line along the front of the trachea and the back of the heart and a second line 1 cm posterior to the anterior margin of the thoracic vertebra. This system does not classify the superior mediastinum as a separate compartment.

For anterior mediastinal masses, the classic differential diagnosis is the “4 Ts”; namely, thymoma, thyroid, teratoma and terrible lymphoma. Further clues may be obtained from the radiographic appearance, the patient’s age and associated clinical manifestations. For example, mediastinal thyroid is rare if there is not demonstrable direct extension from the neck. Calcification, teeth and/or fat in a lesion are in favor of teratoma. Lymphoma is the likeliest diagnosis when there is a multilobular mass. Thymoma often, although not always, overlies the aortopulmonary window.[4]

If a middle mediastinal mass is unrelated to the esophagus, the differential diagnosis includes bronchogenic cyst, lymph node abnormalities (sarcoid, lymphoma, metastases) and vascular lesions. Bronchogenic cyst most often occurs between the carina and the esophagus, but the right lower paratracheal region is not an uncommon alternative location. Among lymph node diseases, tuberculous lymphadenitis has become a more important entity because it is common in patients with acquired immunodeficiency syndrome and frequently involves middle mediastinal lymph nodes. Metastatic disease usually affects lymph nodes in the anterior and/or middle mediastinum. Lung cancer is the most common primary neoplasm involving mediastinal lymph nodes. Most extrathoracic neoplasms do not commonly metastasize to intrathoracic lymph nodes, but there are exceptions. Head and neck tumors, genitourinary neoplasms (especially testicular and renal cell carcinomas), breast carcinoma, gastric carcinoma and melanoma are extrathoracic primary neoplasms with a predilection for hilar and mediastinal lymph nodes.[4]

Posterior mediastinal masses generally represent neurogenic tumors (such as neurofibroma, schwannoma and ganglioneuroma), extramedullary hematopoiesis, hemangioma, infection and vascular lesions.[4] In our case, the mass was located in the middle mediastinum among other mentioned compartments.

Thoracic CT and MRI examinations are quite helpful in better evaluating characteristics of mediastinal masses and lymphadenopathies. The sensitivity and specificity of CT and MRI are almost equal for the evaluation of lymphatic ganglions. No matter what size the lymphatic ganglion is, detection of a central necrosis and extranodal spread is a very meaningful finding for metastasis. CT is a more powerful tool in detecting central necrosis or calcification.[5][6]

Swollen lymph ganglions due to inflammation are called reactive adenopathy, which are seen as oval formations, up to less than 1.5 cm in diameter with a homogeneous content and contrast uptake on CT and MRI images. Central nodal necrosis secondary to intranodal abcess formation may develop in some infections such as staphylococcal infections and is called suppurative adenopathy. Differential diagnosis of suppurative adenopathy and malignant adenopathy is not possible with imaging tool examinations. Multiple lymph ganglions with contrast uptake, central necrosis and globular calcifications are seen radiologically in tuberculous adenitis. Nodal calcifications are mostly seen in tuberculosis, but may also be seen in metastatic papillary thyroid cancer, in lymph ganglions after radiotherapy or in necrotic ganglion abcesses in the healing period. Egg shell calcifications are typically seen in silicosis, sarcoidosis, tuberculosis and amiloidosis.[5]

Lymphadenitis is the most common form of extrapulmonary tuberculosis. The infection often begins in the lungs; therefore, the most common infected sites are the regional lymph nodes, into which the lung parenchyma drains. To understand the course of spreading of the infection one should know the lymph node drains well.

### Mediastinal Lymph Nodes

1-Anterior mediastinal group: They receive afferent vessels from the thymus, thyroid, heart and pericardium, diaphragmatic and mediastinal pleura and middle diaphragmatic nodes.[7]

2-Paratracheal and tracheobronchial groups: These groups receive drainage from most parts of the lungs and bronchi, thoracic trachea, heart and some efferents from the upper para-esophageal nodes of the posterior mediastinal group. The nodes comprising these groups include the upper (station 2R, 2L) and lower (station 4R, 4L) paratracheal, subaortic (aortopulmonary window, station 5), retrotracheal (station 3), and subcarinal (station 7) nodes. The subcarinal nodes are contiguous with the hilar nodes and drain into the paratracheal nodes, preferentially to the right.[7]

3-Posterior mediastinal group: The posterior mediastinal nodes comprises the para-esophageal (station 8) and pulmonary ligament (station 9) nodes. The efferents from the posterior mediastinal nodes.

communicate with the tracheobronchial group, particularly subcarinal nodes and drain chiefly into the thoracic duct, but also drain to the subdiaphragmatic para-aortic or coeliac nodes.[7]

### Lymph Nodes of the Lungs

Lymph nodes are located along the bronchi and may be divided into hilar (station 10R, 10L) and intrapulmonary nodes. The latter consist of interlobar (station 11R, 11L), lobar (station 12R, 12L), segmental (station 13R, 13L), subsegmental (station 14R, 14L) and intraparenchymal intrapulmonary nodes.[7] Most of the lymphatic flow of the lungs is directed toward the interlobar and hilar nodes, which drain into the subcarinal nodes or directly into the lower paratracheal nodes.[7] In our case, subcarinal lymphadenopathy was seen.

During primary tuberculosis infection, bacilli reach from the primary focus in the lung to the nearest lymph nodes (hilar-mediastinal) through the lymphatic way and then to the further lymph nodes. They may also spread hematogeneously to reach other lymph nodes, in which they survive for years as dormant bacilli. Tuberculous lymphadenitis most commonly develop after primary infection, followed by endogenous reactivation of dormant bacilli after years and finally as a result of an infection in the neighborhood.[8] Although mediastinal lymph nodes are the most frequently used primary regional drainage sites, they only form 5% of the reported tuberculous lymphadenitis sites. Important pathologies caused by tuberculous lymphadenitis include compression of the adjacent tissues, caseification and disruption of the lymphadenitis and fibrosis during the healing period of the lymphadenitis.[9]

Successful control of tuberculosis decreases lung tuberculosis cases, but extrapulmonary tuberculosis (EPT) cases do not decrease in a similar ratio.[10] Reported mediastinal tuberculous lymphadenitis cases increase in number, although this is a rare disease in adults. The literature on mediastinal tuberculous lymphadenitis causing dysphagia consists of case reports only. Pimenta et al.[1] reported two cases of adult dysphagia, external compression of the esophagus were detected and surgical diagnosis was made. Rathinam et al.[2] in UK evaluated 14 tuberculous lymphadenitis cases causing dysphagia retrospectively; subcarinal lymph node pathology was detected in seven of them, resembling our case. Park et al.[3] reported a quite large subcarinal lymphadenopathy, similar to our case clinically and radiologically, which caused external compression to the middle portion of the esophagus in a 34-year-old female patient and the diagnosis was made by thoracoscopic biopsy. Popli reported a case and Turner et al. reported two immunosuppressed cases admitted because of dysphagia and fever.[11][12]. Mediastinal tuberculous lymphadenitis should be differentiated from other causes of mediastinal masses. Mediastinoscopy and diagnostic thoracotomy are very efficient in differential diagnosis.[13] Our case was diagnosed by histopathological examination of the material obtained in diagnostic thoracotomy, which was a more appropriate way to reach the lesion. Operative diagnostic thoracic procedures are extremely specific and highly sensitive diagnostic choices in undiagnosed lung, pleural and mediastinal lesions. Thoracotomy is a safe method providing optimal sight for the exploration of the chest cavity and obtaining material if the pericardium, mediastinum, pleura and lungs are concerned.[14]

Treatment with anti-tuberculosis medications is effective and surgery is only needed in the presence of complications.[2] Our case was treated successfully with anti-tuberculosis medications. The importance of this case is the rarity of tuberculosis in adult age in our country manifesting itself only with mediastinal tuberculous lymphadenitis with an atypical presentation of severe dysphagia.
